# Optimal Immunosuppression Strategy in the Sensitized Kidney Transplant Recipient

**DOI:** 10.3390/jcm10163656

**Published:** 2021-08-18

**Authors:** Danae Olaso, Miriam Manook, Dimitrios Moris, Stuart Knechtle, Jean Kwun

**Affiliations:** Duke Transplant Center, Department of Surgery, Duke University School of Medicine, Durham, NC 27710, USA; danae.olaso@duke.edu (D.O.); miriam.manook@duke.edu (M.M.); dimitrios.moris@duke.edu (D.M.)

**Keywords:** sensitization, immunosuppression, induction, B cell, plasma cell

## Abstract

Patients with previous sensitization events against anti-human leukocyte antigens (HLA) often have circulating anti-HLA antibodies. Following organ transplantation, sensitized patients have higher rates of antibody-mediated rejection (AMR) compared to those who are non-sensitized. More stringent donor matching is required for these patients, which results in a reduced donor pool and increased time on the waitlist. Current approaches for sensitized patients focus on reducing preformed antibodies that preclude transplantation; however, this type of desensitization does not modulate the primed immune response in sensitized patients. Thus, an optimized maintenance immunosuppressive regimen is necessary for highly sensitized patients, which may be distinct from non-sensitized patients. In this review, we will discuss the currently available therapeutic options for induction, maintenance, and adjuvant immunosuppression for sensitized patients.

## 1. Immunosuppression in Sensitized Patients

Advancements have led to increased availability and efficacy of immunosuppressive agents, and current 1 year graft survival is 98% with living related donor and 94% for deceased donor kidney transplantation [1]. However, patients with pretransplant positive cytotoxic crossmatch and DSA have shown as high as 70% of graft failure with acute AMR and approximately 50% of grafts loss by 1 year post-transplant [2]. Lefaucheur et al. reported that the incidence of early AMR was 36.4% in patients with an intermediate (MFI 3-6000) level of DSA and 51.3% with a high level of DSA (MFI > 6000) [3]. Immunosuppressive strategies for sensitized patients are largely borrowed from those used in non-sensitized patients. However, variability in outcomes reveals the insufficiency of current immunosuppressive regimens in sensitized patients. Sensitized patients with a negative crossmatch (no donor-specific antibody) showed comparable graft survival to non-sensitized patients in the current organ allocation system [4] even though these patients might have individual center-driven immunosuppressive regimens which are different from non-sensitized patients (i.e., thymoglobulin with higher Tac trough level, etc.). However, immunologically high-risk transplants occurring in sensitized patients, particularly for crossmatch positive, incompatible transplants, require enhanced immunosuppression. Innovation in this field has largely focused on ‘desensitization’ prior to transplantation, or early post-transplant therapies to reduce the risks of acute antibody-mediated rejection (AMR) [5,6,7,8,9,10,11,12,13,14]; however, there has been little examination of the optimal maintenance regimen post-transplant. Furthermore, even with currently available desensitization therapies, both acute AMR and acute cellular rejection (ACR) rates were significantly higher in sensitized/desensitized patients compared to non-sensitized patients [15,16,17]. Recently, changes in deceased donor allocation in the US in particular [18], as well as improvements to living kidney donor sharing schemes [19], have demonstrated that fewer sensitized patients require the need for cross-match positive living transplantation [20]. Nonetheless, patients with pretransplant or de novo donor-specific antibody (DSA) are at greater risk of graft rejection. In this review, we will focus more on maintenance immunosuppression agents in sensitized patients (with positive crossmatches) rather than desensitization strategies even though some treatments can be applied to both indications. Consequently, antibody-targeting strategies such as plasmapheresis (or plasma exchange/immunoadsorption), IVIg, or IdeS (Imlifidase) will not be covered. 

## 2. Choice of Induction Therapy in Sensitized Kidney Transplant Recipients

Induction therapy reduces rates of acute rejection, delayed graft function (DGF), and death after kidney transplantation, and there is a wide variety of induction agents available and used in clinical practice today [21]. Rabbit antithymocyte (rATG) polyclonal antibody or interleukin-2 receptor monoclonal antibodies are the most common agents used for induction in non-sensitized patients. Sensitized patients with preformed HLA antibodies are at greater risk of cellular and humoral rejection, and outcomes can be optimized by using polyclonal induction agents, such as ATG or alemtuzumab, that are associated with a lower risk of rejection and better graft survival [22,23,24,25]. However, the impact of different induction approaches on sensitized patients has not been fully elucidated and the variability in induction therapy can be largely attributed to transplant center choice and clinician preference rather than patient or donor characteristics [23,24,25,26]. 

### 2.1. Basiliximab 

Basiliximab (Simulect) is a non-depleting chimeric anti-CD25 monoclonal antibody against the interleukin-2 (IL-2) receptor on activated T lymphocytes [27]. It is comparable to rATG in patients with low risk of acute rejection, though less effective in high-risk kidney transplant patients, defined as being at risk of DGF or having panel reactive antibody (PRA) > 20% [27,28,29]. Even though activated B cells express CD25 and IL-2 mediated signaling has a critical role for its further differentiation into plasma cells [30], our data in a highly sensitized nonhuman primate model demonstrated a clear limitation of basilliximab in controlling robust memory T and B cell immune responses [31]. Additionally, basiliximab was associated with a greater risk of biopsy-proven acute rejection (BPAR) than rATG in sensitized (HLA class I and II mismatch) kidney transplant recipients without pre-existing DSA [32]. In a study of class I and II HLA DSA-positive, complement-dependent cytotoxicity crossmatch (CDC-XM) negative recipients treated with basiliximab induction therapy, there was a higher incidence of BPAR and AMR [33]. Another study found that DSA against class I and II HLA and high DSA levels, CDC-XM negative, is predictive of early AMR in patients treated with basiliximab induction and triple therapy maintenance immunosuppression [34]. The 5 year graft survival was lowest in patients with class I and II DSA with high binding affinity [34]. Thus, the effectiveness of induction therapy with basiliximab is limited in the sensitized, DSA positive, crossmatch negative patient. 

### 2.2. Thymoglobulin 

Thymoglobulin, or rATG, is a polyclonal gamma immunoglobulin and the preferred choice in sensitized patients at high risk for acute rejection or delayed graft function [21,22]. rATG targets T cells via antibody dependent cytotoxicity (ADCC) and complement dependent cytotoxicity (CDC), but it also depletes B cells and plasma cells since thymus also contains these cell populations. In accordance with this, rATG induces apoptosis of B cells and plasma cells [35]; however, its in vivo efficacy has not been clearly demonstrated [36]. A prospective, randomized, clinical trial showed a decreased incidence and severity of acute rejection when treated with rATG compared to basiliximab at 1 year (16% vs. 26%, *p* = 0.02) and 5 years (15% vs. 27%, *p* = 0.03) post-transplant, but similar incidences of graft loss, DGF, and death [21,34,37]. In moderately sensitized patients (positive DSA and negative flow crossmatch), induction with ATG resulted in reduced occurrence of de novo DSA (dnDSA) and AMR compared to basiliximab [38]. In simultaneous heart and kidney transplants, sensitized patients (with PRA > 10%) treated with rATG induction had lower mortality [39]. Another randomized trial found a significantly reduced incidence as well as severity of acute rejection in high immunological risk patients, defined by Kidney Disease Improving Global Outcomes (KDIGO) as high number of MHC mismatches, younger recipient, older donor, PRA > 0%, presence of DSA, blood group incompatibility, DGF, and cold ischemic time > 24 h, treated with rATG versus basiliximab with acute rejection rates at 1 year (15% vs. 27%, *p* = 0.016) and 5 years (14% vs. 26%, *p* = 0.035) [37,40,41]. Historically, rATG was associated with an increased risk of infection and malignancy compared to basiliximab induction; however, recent studies showed a low overall risk with rATG [37,42,43,44]. A systematic review found a 45% acute rejection risk without ATG induction in patients with calcineurin inhibitor (CI) treatment and 37% acute rejection risk without ATG induction in patients without CI treatment [45]. 

### 2.3. Alemtuzumab

Alemtuzumab is a depleting anti-CD52 antibody that targets T and B cells resulting in lymphocyte depletion and prolonged immunosuppression [46]. Low-dose alemtuzumab is used as an induction agent in sensitized patients undergoing kidney transplantation and, when combined with triple maintenance immunosuppression, is well tolerated and has shown favorable patient and allograft outcomes (death-censored graft survival: 79.2%) [46]. A prospective, open-label, randomized controlled trial found that alemtuzumab induction therapy in sensitized kidney transplant patients is effective and safe with similar rates of acute rejection compared to those treated with rATG [47]. Of interest, an analysis studying the efficacy and safety of induction therapy in non-broadly sensitized kidney transplant recipients (cPRA < 80%) found that alemtuzumab was more effective in decreasing biopsy proven acute rejection than IL-2 receptor antagonists; however, it was also associated with a higher 5 year graft loss risk [48]. A single-center study in sensitized kidney recipients (median PRA 43%, CDC-XM negative) found acceptable patient and graft survival following low-dose alemtuzumab induction [46]. In sensitized pediatric kidney transplant recipients (PRA > 30%), there was equivalent graft and patient survival in those who received intravenous immunoglobulin G (IVIG) and alemtuzumab induction therapy as non-sensitized recipients treated with basiliximab [49]. A prospective study found that the rate of biopsy-confirmed acute rejection in low-risk patients was lower with alemtuzumab when compared with basiliximab, but among high-risk patients, there was no significant difference between alemtuzumab and rATG [50]. However, alemtuzumab is associated with prolonged lymphocyte depletion [46,51] and increased rates of infection [46,52]. 

### 2.4. Rituximab

Rituximab is an anti-CD20 monoclonal antibody that targets B cells, suppresses preformed alloantibodies and reduces peripheral B lymphocytes prior to transplantation [53,54]. A retrospective study of highly sensitized kidney transplant recipients (XM-positive or DSA positive) treated with IVIG and rituximab induction therapy found increased rates of AMR in sensitized recipients compared to low-risk recipients, but similar long-term patient or graft survival at 6 year follow-up [55]. A cohort of seven sensitized patients (mean PRA class I and II were 31% and 51%, respectively) who received rituximab induction therapy showed a reduced occurrence of postoperative humoral rejection [56]. Furthermore, the combination of rituximab with rATG induction therapy in highly sensitized patients (mean class I PRA > 80%) showed superior graft survival at 5 years compared to rATG induction therapy alone (92.9% vs. 48.3%, *p* = 0.02) [24]. Intravenous immunoglobulin and rituximab combined induction therapy in highly sensitized patients (mean PRA = 62%, ≥3 HLA-mismatch, positive FXM or positive pretransplant DSA) was also effective in graft survival, graft function, and overall patient survival [57]. A study of HLA-incompatible recipients (mean cPRA = 80%, repeat HLA mismatches (80%), CDC positive, FCXM positive, or DSA positive) found that rituximab induction reduced the incidence of HLA-antibody rebound (7% with DSA, 33% non-DSA) compared to those transplanted without rituximab (32% DSA, 55% non-DSA), but demonstrated no effect on DSA elimination, AMR, or 5 year allograft survival [58]. Despite rituximab being an acceptable induction therapy, it is not widely used due to safety concerns, such as susceptibility to bacterial infections due to delayed immune reconstitution [59], post-transplant lymphoproliferative disease (PTLD) [60], hypogammaglobulinemia, and progressive multifocal leukoencephalopathy (PML) [61]. Additionally, rituximab therapy can result in a false positive B cell crossmatch when evaluating donor and recipient compatibility for kidney transplant [62].

Although cytolytic induction promotes less graft failure, it should be considered that cytolytic induction may lead to higher cancer risk in patients, such as has previously reported with alemtuzumab for non-Hodgkin lymphoma and rATG for melanoma [42]. Similarly, we have shown significantly prolonged renal graft survival in a sensitized nonhuman primate model when treating with depletional induction compared to basiliximab, but animals receiving depletional induction showed higher levels of infectious complications [31,63,64]. It is also noteworthy that each induction therapy (whether cytolytic or not) is administered according to a standardized dosing regimen that is largely based on experience with non-sensitized patients. Additionally, ATG induction dosing and efficacy was established based on randomized control trials using different maintenance to current practice—either higher Tac trough levels or even cyclosporine based regimen [21,65]. Since the degree of lymphocyte depletion influences later alloimmune responses as well as protective immunity, the dosing and timing of induction agents also requires optimization in sensitized patients.

## 3. Choice of Maintenance Immunosuppression in Sensitized Patients

### 3.1. Triple Immunosuppression

Maintenance immunosuppressive therapy prevents acute rejection and increases allograft survival following kidney transplantation. The standard maintenance regimen varies by center or clinician preference, but the KDIGO Transplant Work Group guideline recommendations include triple therapy immunosuppression consisting of a calcineurin-inhibitor (CNI), such as tacrolimus, an antimetabolite, such as mycophenolate mofetil (MMF), and a glucocorticoid, such as oral prednisone, in kidney transplant recipients [66]. Tacrolimus inhibits activation of T lymphocytes by binding to an intracellular protein, FKBP-12, and inhibiting calcineurin while MMF inhibits T and B cell proliferation. It has been shown that CNIs (cyclosporine and tacrolimus) inhibit antibody production in T and B cell cultures but fail to inhibit immunoglobulin (Ig) production when B cells are cultured with primed T cells [67]. According to the most recent Scientific Registry of Transplant Recipients (SRTR) registry data, over 60% of patients are discharged from the hospital on tacrolimus, MMF, and prednisone triple therapy due to its success as a maintenance immunosuppressive regimen [1,68]. 

### 3.2. Limited Efficacy of Standard Maintenance Immunosuppression in Sensitized Patients 

Triple therapy as maintenance immunosuppressive therapy in non-sensitized kidney transplant patients has high efficacy [68]; however, optimal maintenance immunosuppression in sensitized kidney transplant patients is not well established. Pretransplant DSA (DSA positive, CDC-XM negative) is associated with an increased risk for AMR and poor graft survival [33,69,70,71]. Patients with DSA showed worse kidney allograft survival at 8 years than those without DSA (43 vs. 194 months, *p* = 0.03) and the incidence of AMR in patients with DSA is ninefold higher than in patients without DSA (*p* < 0.001) [72]. AMR is the leading cause of graft loss after kidney transplant, and preformed DSA in sensitized patients can lead to hyperacute rejection, accelerated acute rejection, and early acute AMR [73]. A retrospective single center study found that recipients with DSA, CDC-XM negative had increased rates of acute rejection (54.2% vs. 27.6%), increased AMR (33.3% vs. 19.7%, *p* = 0.176), and increased rates of ACR (16.6% vs. 6.6%, *p* = 0.127) when compared to the non-DSA group [70]. Graft function was similar between groups at 1 year follow-up with no graft loss, and both groups were treated with thymoglobulin induction and standard maintenance triple therapy [70]. A study in which all patients were maintained on standard triple therapy found an increased incidence of acute AMR in patients with pretransplant DSA than those without (41.7% vs. 1.6%, *p* < 0.001) and that higher levels of pretransplant DSA had a detrimental effect on 5 year graft survival [74]. These studies highlight the limitations of standard triple maintenance immunosuppressive therapy and the need for different therapeutic regimens in the sensitized, DSA positive, CDC-crossmatch negative patient population, particularly in light of the experimental evidence highlighted regarding the inability of CNI to prevent antibody production during cognate T–B cell interactions [67].

### 3.3. Replacing Tacrolimus

The mammalian target of rapamycin (mTOR) controls the T cell response (activation and proliferation) and is a valuable immunosuppressant in clinical transplantation. mTOR inhibitors, such as rapamycin (sirolimus) and everolimus, promote the differentiation and function of various helper T cells and suppress the differentiation of memory CD8+ T cells [75]. Furthermore, unlike CNIs, mTOR inhibitors are able to prevent Ig production from B cells when cultured with primed T cells, which suggests their direct impact on B cells [67,76]. Rapamycin has also shown its superiority over tacrolimus with respect to inhibiting B cell to plasma-cell differentiation [77]. In mice sensitized by prior skin graft, preoperative rapamycin increased the expression of regulatory T cells, but did not prolong the survival of mice after cardiac allotransplantation [78]. In donor skin-sensitized mice, those with mTOR deletion in T cells had longer mean survival time (MST 19.5 days) versus wild-type recipients (MST 5.4 days) [75]. Mice sensitized by skin transplant and treated with rapamycin induction therapy were found to have altered frequencies of splenic and intragraft neutrophils, macrophages, and natural killer (NK) cells [79]. A study of rapamycin in sensitized rats found that pretransplant introduction of rapamycin prevented accelerated rejection and prolonged cardiac allograft survival by decreasing the expression of IL-2 mRNA, reducing IL-2 and IFN-gamma cytokine proteins, and modulating the IgM response to prevent class switching and IgG alloantibody production [80,81]. Everolimus treatment led to prolonged graft survival with reduced cell infiltration and prevented tubular atrophy and interstitial fibrosis in sensitized rat recipients; however, chronic allograft nephropathy could not be prevented by everolimus alone in the sensitized recipient [82].

A single-center retrospective analysis of 71 sensitized recipients (cPRA > 50%) showed that mTOR inhibitors (everolimus or sirolimus) had similar outcomes to tacrolimus-based immunosuppressive therapy with MMF [83]. Everolimus was safe and effective in sensitized recipients (PRA > 30%) [84]. Sirolimus and everolimus have been reported to reduce the development of chronic allograft vasculopathy (CAV) most likely due to their antiproliferative and antimigratory effects [85,86,87] as well as by decreasing the de novo DSA [88]. Despite these demonstrated immunologic advantages, the clinical use of mTOR inhibitors lags well behind the use of CNI in transplantation, largely due to unfavorable side-effects of mTOR inhibitors.

## 4. Newly Available Agents for Sensitized Patients

### 4.1. Costimulation Blockade

#### 4.1.1. Belatacept 

Belatacept, a CTLA4-Ig fusion protein that binds to cluster of differentiation (CD) 80 and CD86 receptors on antigen presenting cells (APC), prevents binding to CD28 on T cells, thereby reducing the T cell–dependent immune response [89]. Belatacept has been shown to selectively reduce the humoral response in sensitized, maximally HLA-mismatched non-human primates (NHPs) by suppressing the peripheral and germinal center follicular helper T cell (Tfh) response [90]. Translational studies in highly sensitized NHPs found that desensitization with belatacept in combination with bortezomib or carfilzomib therapy led to significantly reduced AMR, DSA, and plasma cells leading to prolonged graft survival, although it should be noted that these animals received tacrolimus-based triple therapy as maintenance [64,91]. Preliminary studies in animals receiving belatacept in addition to triple therapy indicate a further prolongation of survival, even in a highly sensitized NHP model [92]. In our opinion, these translational studies demonstrate the promising potential of costimulation blockade in sensitized kidney transplant recipients and may be the future of optimal maintenance therapy. Belatacept is associated with 28% less chronic kidney scarring (total 3 studies, 1360 recipients) and better glomerular filtration rate (GFR), blood pressure, and lipid profiles, and lower risk of new onset diabetes compared to CI-treated recipients in the unsensitized recipients, and this was the premise on which it was initially marketed for use. However, the risk of acute rejection, graft loss, and death were comparable [93,94] and despite the similar risk of graft and patient survival and less nephrotoxicity, widespread adoption of belatacept amongst all transplant recipients has been limited by concerns regarding higher rates of acute rejection and PTLD [95,96,97]. Subsequent to this, the understanding of the mechanisms of costimulation blockade resistant rejection (CoBRR) has improved [98,99,100,101]. Additionally, there is developing clinical evidence of significant dampening effects on humoral responses induced by costimulation blockade [90,91,102].

In clinical practice, 163 highly sensitized (cPRA > 98%) kidney transplant patients with pre- and post-transplant belatacept treatment showed a decrease in HLA class I antibodies compared to those who did not receive belatacept, and a clinically significant reduction of post-transplant cPRA, suggesting that belatacept can reduce HLA class I antibody production in highly sensitized recipients [103]. In the BELACOR trial, belatacept (with ATG induction and MMF/steroid maintenance immunosuppression) was given to sensitized patients with preformed DSA (median cPRA = 46%) [104]. Patient and graft survival was 100% and 95.4%, respectively, at 1 year post-transplant and no patient exhibited acute AMR, though there was an increased incidence of acute T cell–mediated rejection [104]. In an effort to reduce the incidence of early T cell mediated rejection, trials of conversion from CNIs to belatacept as maintenance therapy in kidney transplant recipients are associated with significantly improved renal function at 1- and 3 year follow-up, with no increased risk of BPAR or DSA [105,106,107,108]. Similarly, a study of 108 HLA-sensitized kidney transplant patients (PRA > 30% at transplant, history of prior transplant, or positive DSA at time of transplant) converted from CNIs to belatacept showed no difference in rejection-free, patient or graft survival in sensitized versus non-sensitized recipients at 5 years post-transplant. However, rejection-free survival was lower in the sensitized compared to the non-sensitized recipients at 1 year post-transplant, and the average eGFR was also decreased in the sensitized recipients [109].

#### 4.1.2. Anti-CD40mAb

The CD40/CD154 pathway is important for activating T cell differentiation and B cell isotype switching and was found to be important in both the humoral and cell-mediated immunologic response pathways [110]. CD4+ helper T cells are mandatory for generating both naïve and memory DSA responses [111]. Thus, targeting helper T cells in maintenance therapy may lead to decreased AMR and prolonged allograft survival in kidney transplant recipients. Much of the existing evidence is in large animal models as clinical studies blocking the CD40/CD154 pathway have been halted due to the development of thromboembolic complications and direct platelet activation [112,113]. Thromboembolic complications were found to be primarily due to blocking interactions with CD154, which is important for thrombus stability [114]. However, similar events were not observed in antibodies targeting CD40 [115,116,117], so this may be a more promising therapeutic target. A novel blocking, non-depleting Fc-silent anti-CD40 mAb, iscalimab (CFZ533), has been found to prolong renal allograft survival in NHP in the absence of B cell depletion with no evidence of thromboembolic events [118]. Iscalimab blocks primary and memory antibody responses and germinal center formation while preserving peripheral B cells in NHP [119]. Currently, iscalimab is under review in phase II clinical trials in kidney transplantation in the unsensitized patient population. Another anti-CD40 mAb, bleselumab (ASKP1240), is also under phase I/II clinical trial with acceptable outcomes when combined with tacrolimus, as standard therapy [120]. Combination desensitization therapy with anti-CD40 mAb, belatacept, and bortezomib in a sensitized, maximally major histocompatibility complex (MHC)-mismatched, DSA positive, NHP model found prolonged graft survival and decreased risk of graft loss due to AMR, but increased cytomegalovirus (CMV) infections [64]. To date, no studies have looked at the specific indication of anti-CD40mAb in sensitized patients; however, it may offer alternative costimulation blockade with humoral suppressive benefit for sensitized patients.

#### 4.1.3. Anti-CD154mAb

An initial study found that anti-CD154 monoclonal antibodies prevent acute renal allograft rejection in non-sensitized NHPs [121]. A study in donor skin-graft-sensitized mouse recipients of cardiac allografts found that naïve CD8+ T cells depend on CD154 signaling to differentiate into effector T cells, while primed/memory CD8+ T cells remain intact [122]. A study exploring the effects of blocking B7/CD28 and CD40/CD154 costimulatory signals in sensitized mice for allogeneic bone marrow transplant found decreased B cells when blocking B7/CD28 or CD40/CD154 (*p* < 0.01) with a synergistic effect when both signals were blocked (*p* < 0.01), as well as decreased memory and effector T cells when blocking B7/CD28 or CD40/CD154, also with a synergistic effect when both signals were blocked (*p* < 0.01) [123]. This suggests that blocking B7/CD28 or CD40/CD154 costimulatory pathways can inhibit both cellular and humoral immune response with a synergistic effect [123]. Letolizuman/BMS-986004, a novel anti-human CD154 domain antibody that lacks crystallizable fragment (Fc) binding activity, was found to be safe and efficacious with prolonged allograft survival (MST = 103 days) and no evidence of thromboembolism in NHPs [124] and is currently under clinical trial (NCT03605927). A novel anti-CD154 antibody, RD-05, with a genetically modified human IgG4 Fc inhibits B cell activation and antibody formation with no adverse effects of thromboembolism in mice, suggesting clinical applications in AMR as well as antibody-mediated autoimmune diseases including systemic lupus erythematosus (SLE) or idiopathic thrombocytopenic purpura (ITP) [125].

### 4.2. Adjuvant Therapies

#### 4.2.1. Targeting IL-6 or IL-6R

Interleukin (IL)-6 is a pleiotropic cytokine involved in a variety of pathways regulating immune responses, with an important role in the induction of follicular helper T cells which stimulate B cells to differentiate into memory B cells and IgG-secreting plasma cells [126]. The IL-6 receptor (IL-6R) exists as a membrane-bound protein, expressed mostly on hepatocytes and immune cells, and a soluble protein that can bind IL-6 and transmembrane gp130, termed trans-signaling, on nearly all cell types [127,128]. Interactions between IL-6 and IL-6R lead to the activation of transmembrane protein gp130, eliciting signals to downstream JAK and MAPK pathways and the subsequent activation of inflammatory genes [127]. IL-6 is a proinflammatory cytokine that plays a pathologic role in chronic immune disorders, cancer, and transplant rejection [128]. IL-6 also promotes Th17 differentiation and inhibits Treg differentiation, suggesting targeting IL-6/IL-6R may have clinical applications in treating autoimmune disease and organ rejection [127,129]. 

In the sensitized recipient, Chen et al. found that IL-6 deficient mice, sensitized by donor skin transplant, had decreased frequency of effector memory CD4+ and CD8+ T cells after heterotopic cardiac transplant [130]. Mice also had a prolonged MST of 4.2 days compared to WT mice. Shin et al. and Choi et al. studied tocilizumab, an anti-IL-6 receptor monoclonal antibody, as rescue therapy for chronic AMR in HLA-sensitized renal recipients who failed standard treatment [131]. Patients treated with tocilizumab had graft and patient survival rates of 80% and 91% at 6 years post-transplant, compared to graft survival of 50% 2 years post-transplant. Tocilizumab therapy also caused significant reductions in DSA at 2 years post-transplant. Four patients experienced graft loss approximately 6 months after cessation of tocilizumab, suggesting IL-6/IL-6R pathway blockade may be important in developing targeted rescue or maintenance immunosuppressive therapy. Tocilizumab treatment decreased total IgG and IgG1-3 subclasses, suppressing immunoglobulin production in B cells and treating chronic AMR in HLA-sensitized recipients [132]. Other IL-6/IL-6R blockades that are FDA-approved or undergoing RCT for autoimmune diseases include clazakizumab, siltuximab, sarilumab, olokizumab, sirukumab, and tofacitnib [127]. Presently, a phase II RCT studying clazakizumab, a genetically engineered humanized monoclonal antibody against IL-6, in late AMR (NCT03444103) is underway, although the indication is treatment of AMR occurring post-transplant, rather than maintenance or preventative therapy in the sensitized recipient.

#### 4.2.2. Anti-BAFF 

B cell activating factor (BAFF, also known as BLyS) and a proliferating inducing ligand (APRIL) are cytokines that belong to the tumor necrosis factor family whose primary function is to enhance B cell survival and differentiation into plasma cells [133]. Both are currently used in treating autoimmune diseases such as systemic lupus erythematosus (SLE) and Sjogren’s, but several studies have found that high levels of serum BAFF are associated with the formation of anti-HLA DSA, increased risk of AMR, and poor renal graft survival [134,135,136,137,138]. BAFF is highly expressed in the membrane and renal tubule epithelial cells of transplanted kidneys with acute rejection, and high pretransplant BAFF has been found to predict risk of graft rejection [139].

Belimumab is a human antibody that binds soluble and membrane-bound BAFF, and is FDA-approved for the treatment of SLE [140]. Stohl et al. found that belimumab decreased IgG autoantibodies, naïve and activated B cells, and plasma cells, but did not reduce memory B or T cells in patients with SLE [141]. Banham et al. conducted a phase II trial studying belimumab infusions plus standard of care (basiliximab, mycophenolate mofetil, tacrolimus, and prednisolone) immunosuppression therapy in adult kidney transplant patients (NCT01536379) [142]. They found similar proportions of adverse events between treatment and placebo groups, and no significant reduction in naïve B cells at 6 months post-transplant. Secondary endpoints found that belimumab-treated patients had decreased production of de novo non-HLA antibody, fewer memory B cells, and increased IL-10 versus IL-6 production. This suggests that belimumab may lead to a reduction in antibody-forming cells and dampen the humoral response; however, it should be noted that the patient cohort was not sensitized. Steines et al. studied the effects of anti-BAFF antibody in a rat renal transplant model [143]. They injected anti-BAFF antibody after allogeneic kidney transplantation and found that anti-BAFF treatment reduced the humoral response, including reduced splenic IgG expression, naïve B cells, plasma cells, and expression of IL-6, CD40 and inducible T cell costimulatory ligand. 

In the sensitized recipient, translational pre-clinical studies of anti-BAFF agents have shown promising effects as a potential post-transplant immunosuppressive therapy, although this has not been examined in humans. Our group has studied atacicept, a recombinant fusion protein also known as TACI-Ig, in NHP kidney allotransplantation using maximally MHC class I and II mismatched donors [144]. The results show a decreased humoral response and prolonged graft survival. The atacicept-treated group had reduced early de novo DSA production and reduced B cell repopulation, suggesting that atacicept may play a role in reducing the germinal center or pre-germinal center B cell response. Bath et al. studied an APRIL/BLyS blockade, TACI-Ig, as desensitization and maintenance treatment in an MHC mismatched, sensitized rodent kidney transplant model [145]. They found that TACI-Ig depleted plasma cells, IgG, and IgM secreting cells, as well as decreased incidence of AMR, but did not decrease post-transplant DSA levels. Animals treated with TACI-Ig alone as desensitization therapy experienced increased rates of AMR, noting that the timing of B-lymphocyte depletion plays an important role in AMR. Additional studies in a chronic rejection kidney transplant model showed that TACI-Ig significantly decreased B lymphocytes, antibody production, and splenic germinal center formation in a sensitized mouse model, but increased T lymphocytes, specifically effector T lymphocytes [146]. Other anti-BAFF antibodies undergoing clinical trials, tabalumab, blisibimod, and BR3-Fc, have not been studied as maintenance immunosuppression therapies in kidney transplantation, although attempts to desensitize using tabalumab alone pretransplant have not demonstrated efficacy [147].

#### 4.2.3. Targeting PC

##### Conventional PI

Bortezomib was the first proteasome inhibitor (PI) to be FDA-approved for the treatment of malignant plasma cell diseases [148]. Proteasome inhibitors, including bortezomib, carfilzomib, oprozomib (ONX 0912), and ONX 0914 (immunoproteasome inhibitor), reduce proliferating B cells and antibody production conceivably by inducing apoptosis of activated B cells [149]. Bortezomib has been shown to be effective in preventing AMR and ACR, as well as reducing DSA in kidney transplant recipients [150]. Since then, bortezomib has been studied in combination therapy with plasmapheresis (PP) and IVIG with or without rituximab or steroid as plasma cell–targeted therapy in sensitized kidney transplant recipients, showing success in reducing DSA, treating acute or late AMR [151,152] as rescue or primary treatment [153,154,155], and reducing plasma cell rich acute rejection [156,157]. Recently, six patients who developed acute AMR received bortezomib and belatacept combination therapy, which led to the reversal of AMR and reduction in circulating DSA [158]. The response to PI therapy differs between early and late AMR, with optimal clinical responses seen when bortezomib is used to treat early AMR [159], while a subsequent clinical trial indicated a lack of efficacy of bortezomib in treating late AMR [160]. In the sensitized setting, pretransplant desensitization with both costimulation blockade and bortezomib significantly prolonged graft survival and reduced the risk of AMR in a NHP model [64]. Additional desensitization studies utilizing bortezomib and costimulation blockade (belatacept and anti-CD40 mAb) as dual targeting therapy of plasma cells and the germinal center response led to prolonged graft survival despite significant CMV infection and drug toxicity [64]. Most recently, carfilzomib, a second-generation PI, has been studied for desensitization, showing reduction of preformed HLA antibodies (median reduction of 72.8%) with a plasma cell reduction in bone marrow (69.2% reduction) and acceptable drug safety and toxicity [161]. As described, PIs have been studied mostly in the setting of desensitization therapy, but there is a growing interest in utilizing PI as maintenance immunosuppression or treatment for AMR.

## 5. Possible Limitations of Combined B and T Cell Suppression

The current clinical practice for managing immunosuppressive agents in sensitized patients is largely based on conventional risk management of transplant patients. Sensitized patients (considered high-risk patients) are mainly treated with a conventional strategy using CI plus MMF, avoiding minimization. However, this patient population continues to show worse graft survival than the unsensitized patient population. Although adjuvant therapies such as costimulation blockade have potential positive benefits, it is hard to justify that any single agent introduced with conventional immunosuppressive regimens can successfully prevent rebound or de novo DSA production in sensitized patients. Considering the high-risk immunological profile of sensitized patients, the combination of conventional immunosuppression with adjuvant treatments could be a solution. Recent data regarding combined tacrolimus or rapamycin with belatacept in non-sensitized patients displayed promising results. The acute rejection (AR) rate at 3 months post-transplant was significantly reduced in basiliximab induction with belatacept and tacrolimus/MMF/steroid (15%) compared to belatacept maintenance (38%). After tacrolimus/steroid discontinuation, the 12-month AR rate was lower in the belatacept/tacrolimus group (33%) compared to belatacept alone (50.5%) treatment group. However, the AR rate at 12 months was significantly higher than the tacrolimus alone treatment group. Interestingly, the extended tacrolimus treatment (up to 9 months and discontinuation at 11 months post-transplant) together with belatacept showed similar 12-month AR rates without risk of CMV or BK virus reactivation compared to the tacrolimus alone treatment group [162]. Belatacept plus rapamycin also exhibited a lower AR rate (4%) at 12 months compared to belatacept/MMF (15%), and a comparable rejection rate to tacrolimus/MMF (3%) with rATG [163]. Furthermore, belatacept/rapamycin maintenance immunosuppression in conjunction with alemtuzumab induction therapy displayed 95% 5 year post-transplant graft survival with 100% patient survival [164]. However, optimizing the combination of these agents in sensitized patients will require further and specific clinical trials to identify the best combinations to use in clinical practice. It is also important to not only confirm the mode of action of the additional agents (i.e., suppression of DSA production) but also to identify any unexpected or unwanted side effects (i.e., increased risk of infections, etc.). Combining these agents may promote overimmunosuppression and increase the incidence of opportunistic infections or PTLD in these patients. Therefore, large animal models, such as the use of sensitized NHPs, provide a useful platform to further evaluate the best combined approach, the timing of treatment (or weaning) of additional agents, the durability (or duration) of the treatment and the associated risks before translating results into clinical practice. This also need to be accompanied by close surveillance with proper monitoring tools which reflect recipient alloimmune status and protective immunity.

## 6. Concluding Remarks

Conventional maintenance immunosuppression with tacrolimus, MMF, and steroids after lymphocytic depletion has been widely used for managing sensitized patients with incompatible organ transplants considered to be at increased immunological risk of rejection. Despite the fact that these T cell centric approaches are effective in non-sensitized patients, they fail to control the post-transplant humoral response of sensitized patients. Given the known challenges of the primed immune system of the sensitized patient, targeting B cell or T cell interactions with B cells should be considered as part of an optimal maintenance immunosuppression for this patient population. Due to the rapid evolution of agents targeting individual steps of humoral responses, as well as advances in our understanding of AMR, it is possible to design a mechanistically rational approach for the sensitized patient.
